# Dynamic Responses of Selective Brain White Matter Fiber Tracts to Binge Alcohol and Recovery in the Rat

**DOI:** 10.1371/journal.pone.0124885

**Published:** 2015-04-20

**Authors:** Adolf Pfefferbaum, Natalie M. Zahr, Dirk Mayer, Torsten Rohlfing, Edith V. Sullivan

**Affiliations:** 1 Neuroscience Program, SRI International, Menlo Park, CA, United States of America; 2 Department of Psychiatry and Behavioral Sciences, Stanford University School of Medicine, Stanford, CA, United States of America; 3 Department of Diagnostic Radiology, University of Maryland School of Medicine, Baltimore, MD, United States of America; Italian Institute of Technology, ITALY

## Abstract

To determine the dynamics of white matter vulnerability to excessive alcohol consumption, diffusion tensor imaging (DTI) was used in an animal model of alcohol exposure. Quantitative, *in vivo* fiber tracking results are presented from rats with DTI conducted at 3 time points: baseline; after 4 days of intragastric alcohol to blood alcohol levels of ~250mg/dL; and after one week of recovery. Binge alcohol followed by a week of sobriety resulted in rapidly reversible decreases in fractional anisotropy (FA), a measure of the coherence of fiber tracts, in callosal genu and fimbria-fornix but not splenium; and increases in mean diffusivity (MD), an index of freely diffusing water in tissue, selective to the fimbria-fornix. These effects were confirmed with tract-based spatial statistics (TBSS). The directionality of changes in DTI metrics reproduce those observed in human alcoholism. That a single exposure to binge alcohol can cause substantial transient changes detectable in DTI metrics demonstrates the potential for rapid neuroplasticity.

## Introduction

Postmortem studies of human cases of alcohol use disorders (AUD) suggest that brain volume reductions are largely accounted for by shrinkage of white matter [1–3]; for example, the corpus callosum is significantly thinned [3–5]. In vivo, disruption of white matter integrity in chronic alcoholism can be detected with diffusion tensor imaging (DTI) [6–10] and can be more sensitive than measures of macrostructure (i.e., white matter volume) for detecting the throes of alcoholism [11,12].

Metrics of DTI provide indices of different features of fiber condition: fractional anisotropy (FA) indexes the degree of coherence of fiber tracts and ranges between 0 (lowest) to 1 (perfect linearity), and mean diffusivity (MD) is indicative of unrestricted water movement and reflects some aspects of tissue architecture [13–18]. Two derivative measures of mean diffusivity provide indices of selective features of white matter tracts: longitudinal diffusivity (also called axial, λ_L_) is thought to reflect axonal integrity, whereas transverse diffusivity (also called radial, λ_T_) is thought to reflect myelin integrity [19–21]. A common finding in adult human alcoholism is evidence for greater degradation (lower FA and higher MD with λT particularly affected)[22] of anterior (e.g., genu, anterior cingulate bundle, and frontal forceps) than posterior (e.g., splenium) white matter fiber tracts [7,8,11,23–27]. Mechanisms of alcoholic white matter injury, however, remain unknown.

Animal studies permit the interrogation of factors underlying white matter sensitivity to alcohol exposure. Animal models of components of human AUD enable control over relevant variables, such as amount, length, and pattern of alcohol exposure [28,29]. A variety of methods are available for exposing animals to alcohol and include vapor inhalation, intragastric infusion, and voluntary drinking. The 4-day binge protocol has been used extensively to model alcoholism in rodents because sustained blood alcohol level (BAL) elevations by intragastric alcohol administration have been shown as adequate for rapid induction of physical dependence in the rat [e.g., 30,31].

Although other studies have examined white matter tracts in rodents [32,33], to our knowledge, only one study measured DTI metrics in response to alcohol exposure in the rat: a single intragastric dose of alcohol resulted in mild decreases in the apparent diffusion coefficient (ADC, a DTI metric similar to MD) and no effects on FA [34]. This decrease in ADC is distinct from typical findings in humans with chronic alcoholism [8,24–27] and may be due to a species (rat vs. human) or alcohol exposure (acute vs. chronic) effects. To expand on this previous DTI finding in a more typically implemented animal model of alcohol exposure, the present study performed *in vivo*, quantitative fiber tracking on DTI data acquired in rats exposed to short-term binge alcohol *via* intragastric gavage [35]. We tested two principal hypotheses: 1) *in vivo* DTI in the rat would detect selective changes to white matter in response to binge alcohol exposure with greater abnormalities (lower FA and higher MD) in anterior than posterior fiber systems; and 2) as observed for ventricular volume and metabolite changes [35], disruption of fiber tracts would be transient, showing normalization with one week of recovery from alcohol. To minimize bias in selecting a limited number of white matter tracts to quantify, we also analyzed data using tract-based spatial statistics (TBSS), which determines DTI metrics in the whole white-matter skeleton of the brain.

## Materials and Methods

### Ethics Statement

The Institutional Animal Care and Use Committees at SRI International (protocol #01019) and Stanford University (protocol #8800) approved all procedures.

### Alcohol Treatment and Schedule

**Fig 1:**
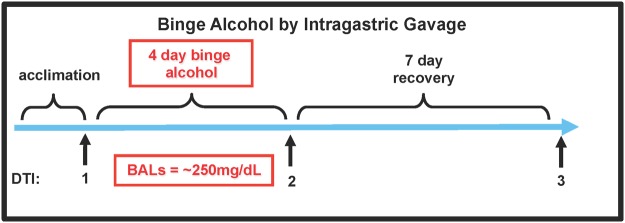
Schedule of alcohol exposure.

The study group initially included 22 wild-type, male Wistar rats (Charles River Laboratories) weighing 334.83±3.7g at baseline, singly housed with free access to food and water, with lights on for 12 hours starting at 8:00. DTI was conducted at 3 sequential magnetic resonance (MR) scanning sessions: DTI 1—pre-treatment baseline; DTI 2—following 4 days of intragastric gavage of alcohol or dextrose (control); and DTI 3—following one week of recovery, that is, without alcohol (Fig 1). This same group of rats underwent examination with structural MR imaging and MR spectroscopy at the same MR scanning sessions [35].

After baseline scanning, 11 rats were assigned to the alcohol group and received an initial “loading” dose of 5g/kg 25% alcohol w/v *via* oral gavage, then a maximum of 3g/kg every 8 hours for 4 days. On each of the 4 days, animals were weighed and tail vein blood samples were collected to determine BALs in plasma assayed for alcohol content based on direct reaction with the enzyme alcohol oxidase (Analox Instruments Ltd., UK). Target BALs were 250mg/dL because alcohol dependent individuals show normal motor function at fairly high alcohol levels (200–400mg/dL) [36,37]. Alcohol was administered according to body weight, BALs, and behavioral signs of intoxication. Dosing was adjusted for each animal so that less than 3g/kg was administered if the animal’s behavior or BALs indicated that it was still too intoxicated from the previous dose to receive the full 3g/kg dose. Specifically, doses were determined individually for each animal according to the published schedule described by correlations between BALs and severity of intoxication [38]: the greater the severity of intoxication, the smaller the dose of alcohol administered [39]. Daily doses averaged 7.21±.32g/kg/animal (cumulative dose across 4 days = 28.85±1.26g/kg/animal) at a daily volume averaging 8.87±.46mL/animal. Control animals received volumes of 5% dextrose equivalent to 3g/kg alcohol (daily volume averaging 11.86±.43mL/animal) at comparable times to the experimental animals, i.e., ~7:00, 15:00, and 23:00. The alcohol dose was titrated based on BALs and behavioral intoxication states, whereas dextrose was given at a constant volume based on 3g/kg alcohol per day; thus the control group received a greater fluid load than the alcohol group (t(19) = 4.75, p = .0001). These methods resulted in average BALs of ~250mg/dL in the alcohol-treated rats. Two alcohol and one control rat received baseline and binge scans but died before the recovery scans.

### MRI Acquisition

Following our established protocol [35,40,41], each animal was held in an MR-invisible structure, which provided support for the radiofrequency (RF) coil and a nose cone for the delivery of isoflurane anesthesia (1–2%) and oxygen (~1.5 l/min). Rectal temperature and oxygen saturation from the hind limb were monitored throughout the duration of the 2-hour MR acquisition. A pad of warmed saline placed under the animal provided heating.

The experiments were conducted on a clinical 3T GE Signa human MR scanner equipped with a high-strength insert gradient coil (peak strength = 600 mT/m; peak slew rate = 3200 T/m/s) [41–43]. As previously described [44], the gradient system was operated at a maximum amplitude of 500 mT/m with a slew rate of 1800 mT/m/ms to minimize vibration and acoustic problems. A custom-made rat brain quadrature head coil (∅ = 44 mm) was used for both RF excitation and signal reception.

A gradient recalled echo (GRE) localizer scan (echo time (TE)/repetition time (TR) = 2.1/24.6 ms, flip angle = 30°, field of view (FOV) = 80×80 mm^2^, 256×128 matrix, three 5-mm slices per plane, scan time = 47 s) was used to position the animals in the scanner and for graphical prescription of the fast spin-echo (FSE). Dual-echo, FSE images were acquired in the axial plane (coronal to the magnet system bore; TE1/TE2/TR = 11.3/56.7/7000 ms, FOV = 60×60 mm^2^, 256x256 matrix, echo train length of 8, or 26 contiguous slices, 0.7mm thick). The FSE data were used for the prescription of the DTI acquisition sequence.

The RF and gradient scheme of the implemented DTI echo-planar pulse sequence (FOV = 32×32 mm^2^, TE/TR = 32.8/2000 ms) was previously published [44]. Multi-slice data were acquired in axial orientation (magnet coronal) at in-plane resolution of 0.5 mm and through-plane resolution 0.7 mm, with and without diffusion weighting. A partial k-space acquisition scheme (n_read_ = 64, n_phase_ = 48) was applied to reduce TE. Frequency encoding direction was left to right. Data were acquired with a readout bandwidth of ±200 kHz (G_read_ = 147 mT/m) and an echo spacing of 0.544 ms for a total readout duration of 26.1 ms. Symmetric diffusion-weighting gradients were applied with a b-value = 1464 s/mm^2^ in 6 noncollinear directions (+x+y, +y+z, +x+z, -x+y, -y+z, +x-z); the same 6 directions with opposite polarity were acquired to compensate for the cross-terms caused by both imaging and crusher gradients [45]. Six directions are the minimum needed to define the diffusion tensor, and we chose to enhance single-shot signal-to-noise ratios with multiple averages of the same direction rather than using more directions with fewer averages. Crusher gradients to dephase transverse magnetization resulting from imperfect refocusing pulses were applied in all 3 directions immediately before and after the 180° pulse. Frequency-selective lipid suppression and outer-volume suppression modules preceded the imaging sequence. Saturation bands were placed around the FOV. Six averages per acquisition were collected with the positive and negative polarity gradient scheme and repeated 12 times for a total DTI acquisition time of 45 min.

### DTI Analysis

DTI quantification was preceded by eddy-current correction on a slice-by-slice basis using within-slice registration, which takes advantage of the symmetry of the opposing polarity acquisition [46]. The reversing diffusion gradient polarity scheme also eliminates the need to account for the cross terms between imaging and diffusion gradients by averaging the opposite polarity data [45], reducing the data to six non-collinear diffusion-weighted images per slice. Using the field maps, B0-field inhomogeneity-induced geometric distortion in the eddy-current corrected images was attenuated with PRELUDE (Phase Region Expanding Labeller for Unwrapping Discrete Estimates)[47] and FUGUE (FMRIB's Utility for Geometrically Unwarping EPIs; http://www.fmrib.ox.ac.uk/fsl/fugue/), although this procedure had minimal effect on the data. The basis images were interpolated to .125 mm isotropic resolution with a windowed sinc in-plane and linear through-plane function and smoothed with a 3×3×3 box-car average. DTI metrics (FA, MD, longitudinal diffusivity [λ_L_], and transverse diffusivity [λ_T_]) were computed with standard tensor fitting methods [17].

### Fiber Tracking

The genu, splenium, and bilateral fimbria-fornix tracts of the hippocampus were identified as point targets in the FSE image of a laboratory standard animal, which was selected based on image quality (e.g., low image noise, no visible artifacts) and animal positioning (e.g., centered within the available FOV with no cropping). The baseline late-echo FSE anatomical images were registered nonrigidly [48](http://nitrc.org/projects/cmtk) to the standard animal image. We used the Normalized Cross-Correlation similarity measure to drive the registration, which used a three-level B-spline transformation [49] with a final control point spacing of approximately 0.7 mm (the exact control point spacing is a function of the initial control point spacing, here 2.5 mm, the number of grid refinement steps, here 2, and the image field-of-view).

For each follow-up session, each animal's image FSE was also registered nonrigidly to the same animal's baseline FSE image. The target point locations were then mapped from the standard animal space into native space for each animal and session using the aforementioned registration to the standard animal space (for the baseline session) or the concatenation of the standard-to-baseline with the baseline-to-follow-up registration. For longitudinal studies we have found that, while there is likely a small increase in absolute registration error due to concatenation, a sequential approach significantly reduces the inconsistency of quantification across time points [50].

**Fig 2:**
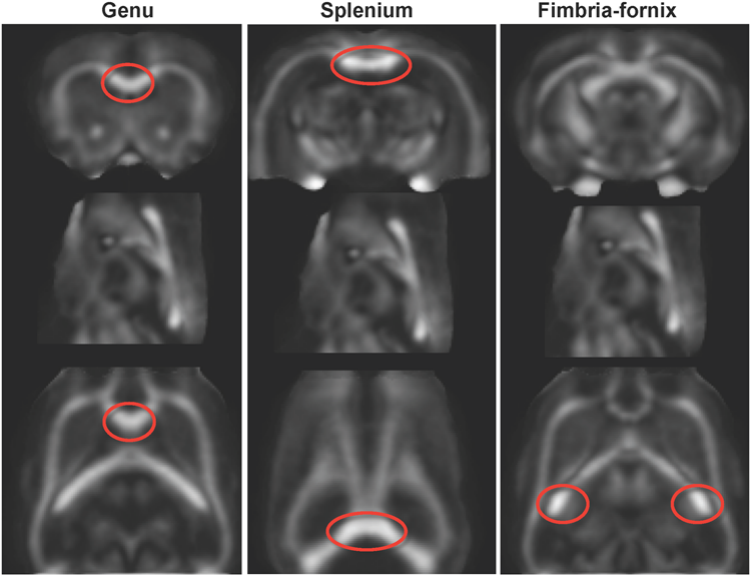
FA maps with circles indicating regions of the genu (right) and splenium (middle) of the corpus callosum and fimbria-fornix (left) used for fiber tract quantification of FA and MD.

To improve the precision of target locations, an automated 5-point, 3-dimensional local maximum search within the FA image ensured location of the target within the center of the given fiber track. The identified point was then expanded to a target, which was a 7 x 7 plane, 3-voxels thick, perpendicular to fiber orientation. Thresholds used included an FA value limit of .17 and an angle maximum of 37°. Tracking sources were automatically defined as planes parallel to the genu and splenium targets and anterior/posterior to the fimbria-fornix. Quantitative fiber tracking routines and parameters, developed by Mori and Xu [51,52] and distributed by G. Gerig [53] (www.ia.unc.edu/dev/download/fibertracking), were used and produced pictorial fiber bundle representations. Quantification of the mean FA and MD of the fibers were similar to those we have previously used *in vivo* in the human brain [7,54] and rat brain [44]. Mean FA and MD were calculated for fibers of the genu and splenium of the corpus callosum, and left and right fimbria-fornix tracts (Fig 2).

### Tract Based Spatial Statistics (TBSS)

For TBSS analysis, the structural registrations to the aforementioned laboratory standard were used to reformat each animal's FA image into common space. As described above for tracking target transformation, we used concatenated standard-to-baseline and baseline-to-follow-up registrations to reformat the FA image from the follow-up images of all animals, again to reduce the longitudinal inconsistency the registrations. Initial mean FA image and FA skeleton were constructed, with the skeleton extraction threshold set at FA = 0.20. Contrasts were performed for alcohol vs. control (dextrose) at DTI 2. Statistical testing was performed using TFCE (Threshold-Free Cluster Enhancement) [55] with 1000 permutations. The resultant clusters were examined for voxels meeting p≤.05 fully corrected for multiple comparisons across space.

### Statistical Analysis

Principal differences between alcohol-exposed and control groups were tested with repeated-measures analysis of variance (ANOVA) across imaging sessions, where group (i.e., alcohol or control)-by-session (i.e., DTI 1, DTI 2, DTI 3) interactions would provide support for potential effects of alcohol treatment. ANOVAs were conducted using lme in R (http://www.r-project.org/) for data from rats that completed at least the first two DTI sessions (control = 10, alcohol = 9; two alcohol and one control rat had only DTI 1 and DTI 2 data available for analysis). The lme allowed the ANOVA to be calculated using all data and accounting for empty cells (i.e., missed sessions). Follow-up t-tests were employed where needed with appropriate adjustment for multiple comparisons. Relations between variables were tested with Pearson correlations and verified with nonparametric Spearman rank-order correlations because of the small samples.

## Results

**Fig 3:**
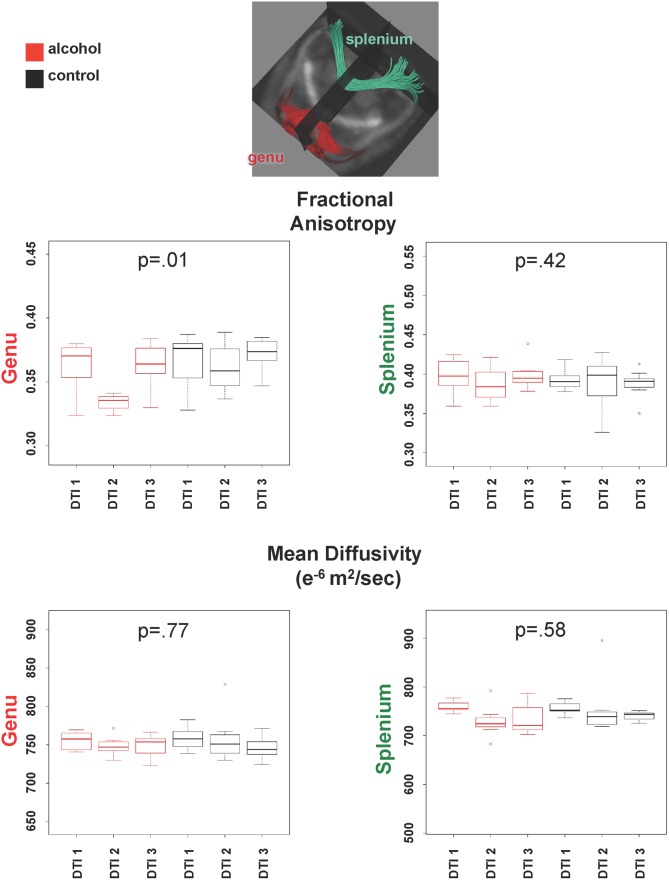
Inset shows fiber tracts of the genu (red) and splenium (green). Graphs show quantified results of FA (top) and MD (bottom) in corpus callosum genu and splenium. Dots here an in consequent graphs represent outliers.

**Fig 4:**
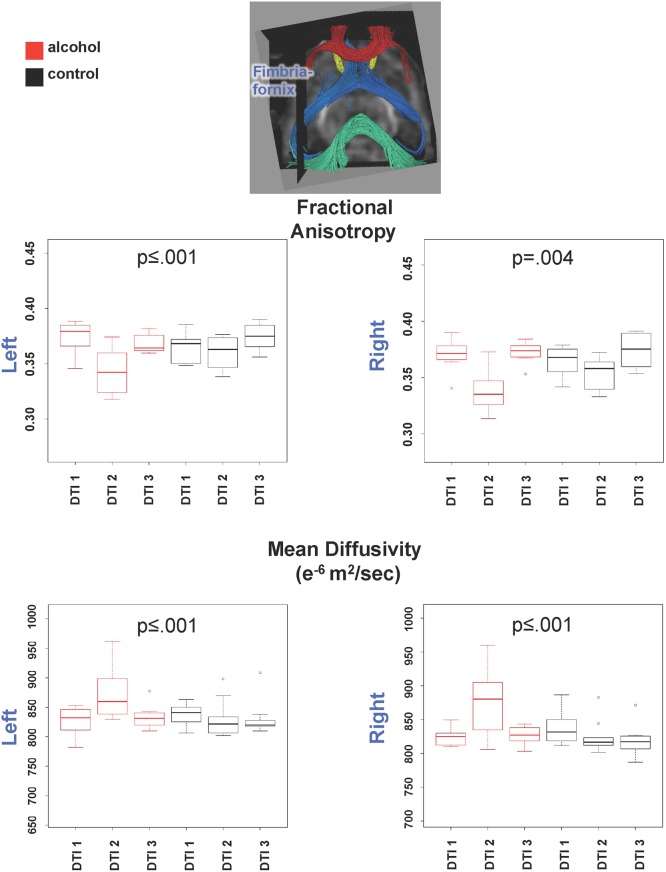
Inset shows fiber tracts of the fimbria-fornix (blue). Graphs show quantified results of FA (top) and MD (bottom) in left and right fimbria fornix.

Repeated-measures ANOVAs for FA of each fiber track yielded group-by-session interactions in the genu (F(2,55) = 4.95, p = .010) but not the splenium (F(2,55) = .88, p = .42) (Fig 3). Interactions were also significant for both the left (F(2,55) = 7.91, p = .0009) and right (F(2,55) = 6.09, p = .004) fimbria-fornix tract (Fig 4). In all cases, paired t-tests indicated that the interaction was attributable to a significant drop in FA from the first to the second DTI session followed by a return in FA, observed in the alcohol but not the control group. For MD, the alcohol effect, identified as group-by-session interactions, was limited to the fimbria-fornix: left (F(2,55) = 8.71, p = .0005) and right (F(2,55) = 11.40, p = .00007). In complement to FA, the interaction indicated a rise in MD followed by a return to baseline in the alcohol-treated group only. One control animal had an exceptionally high MD at DTI 2, but the results were the same with or without its data in the analysis. ANOVAs based on data from animals that completed two or more DTI sessions yielded the same results as the ANOVAs based on rats with complete sessions.

**Fig 5:**
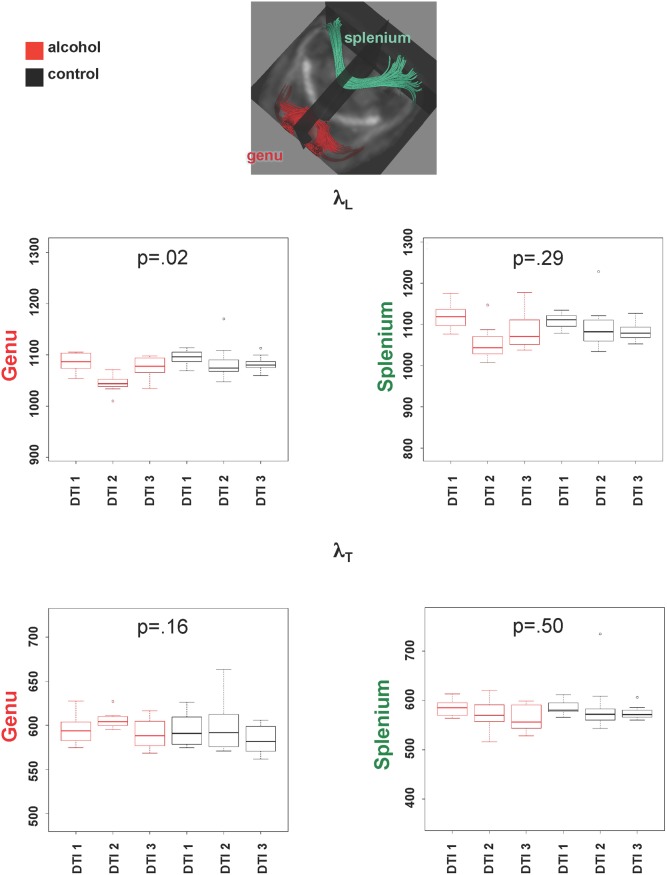
Graphs show quantified results of λ_L_ (top) and λ_T_ (bottom) in corpus callosum genu and splenium.

**Fig 6:**
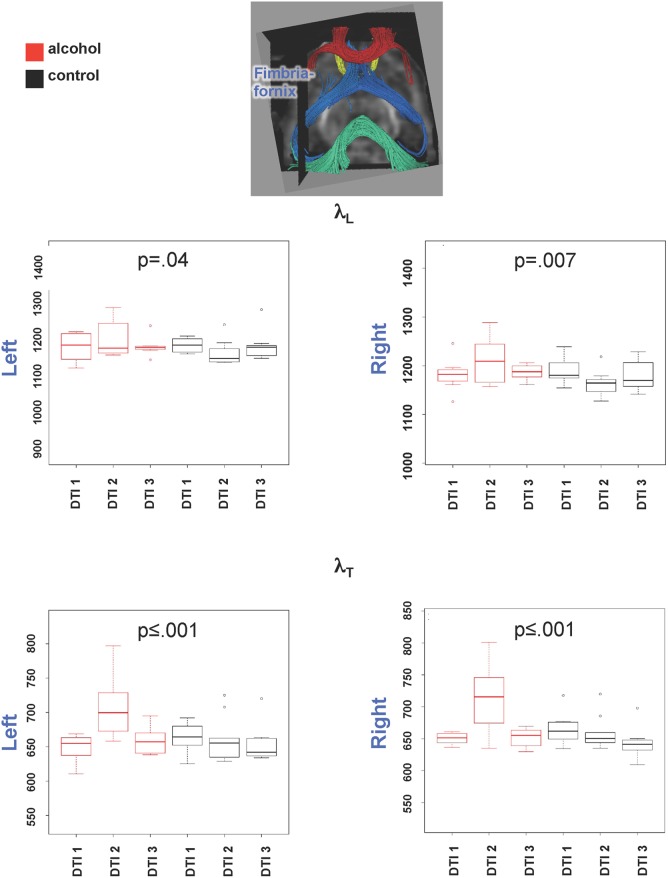
Graphs show quantified results of λ_L_ (top) and λ_T_ (bottom) in left and right fimbria fornix.

Two-group-by-three session interactions were also significant for λ_L_ (F(2,55) = 4.44, p = .02) but not λ_T_ (F(2,55) = 1.88, p = .16) in genu (Fig 5). Here the pattern of changes were similar to FA, that is, attributable to a drop in λ_L_ from DTI 1 to DTI 2, followed by a return of λ_L_ to baseline levels at DTI 3, in the alcohol but not control group. Significant group-by-session interactions were noted for both λ_L_ (left: F(2,55) = 3.42, p = .04; right: (F(2,55) = 5.45, p = .007) and λ_T_ (left: F(2,55) = 10.94, p = .0001; right: (F(2,55) = 12.5, p = .00003) in fimbria-fornix, but followed the MD pattern, that is, both were elevated at DTI 2 but returned to baseline at DTI 3 in the alcohol-treated group only (Fig 6). The same patterns were present in the groups including animals that only completed the first two scanning sessions.

### TBSS Results

**Fig 7:**
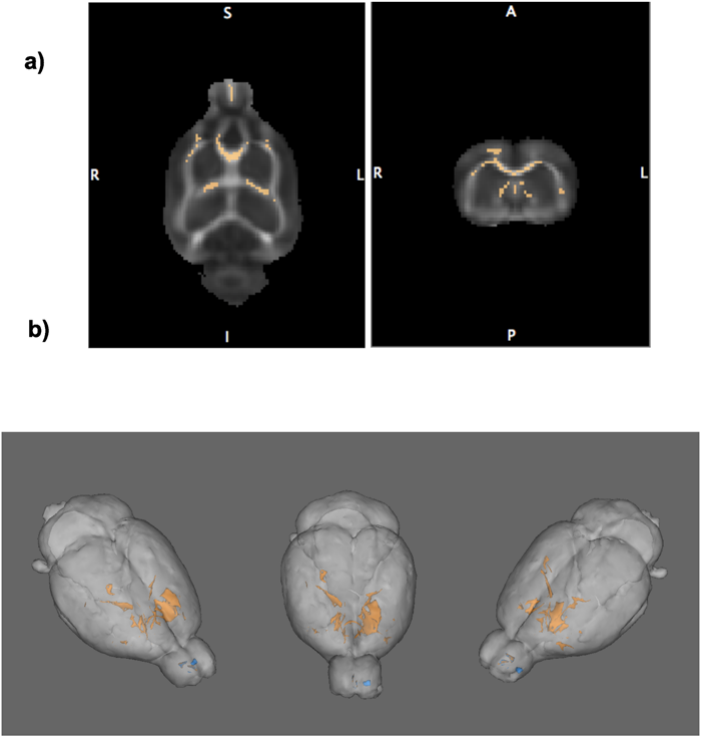
a) Axial and coronal FSL presentations of a rat white matter skeleton indicating voxels with lower FA in alcohol-exposed than dextrose-treated animals after acute binge treatment (i.e., DTI 2). b) TBSS results in 3D at DTI 2 (orange) show where FA was lower in the alcohol than dextrose treated animals at p<.02. In blue are indicated the voxels showing FA differences between alcohol-exposed than dextrose-treated animals at DTI 3.

Fig 7a shows axial and coronal TBSS maps demonstrating white matter skeleton voxels (in orange) with lower FA in alcohol-exposed than in dextrose-treated animals at DTI 2. At DTI 3, the alcohol-treated rats had ~900 voxels with lower FA than the dextrose-treated control rats at p<.02; ~850 voxels differed at p<.001. Most voxels with lower FA at DTI 2 were in the genu and lateral extent of the corpus callosum, the fimbria-fornix and frontal forceps (Fig 7b, in orange). No voxel met p<.05 criterion for opposite contrast of lower FA in dextrose- than alcohol- exposed animals. A comparison between alcohol- and dextrose- treated animals at DTI 3 showed minimal FA differences (at p<.05 family-wise error corrected, Fig 7b, in blue).

### DTI findings and alcohol levels

**Fig 8:**
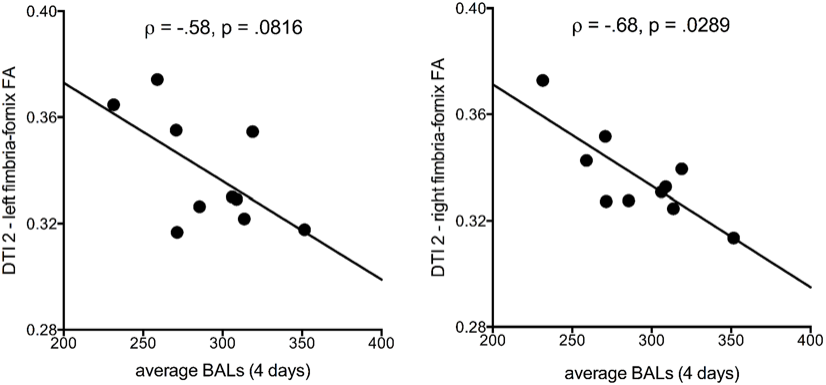
Correlations between left and right fimbria-fornix FA and average BALs across the 4 days of binge alcohol treatment. Bilateral results were also significant (Rho = -.65, p = .0425).

To examine the effect of the full extent of the alcohol binge treatment on the DTI metrics, we tested the relation between DTI metrics and average BALs attained during the 4-day binge. None of the correlations for genu or splenium FA or MD was significant. By contrast, for the fimbria-fornix, higher average BALs obtained during binge sessions correlated modestly with lower FA at DTI 2 (bilateral: Rho = -.65, p = .04; right: Rho = -.68, p = .03, left: Rho = -.58, p = .08) (Fig 8).

## Discussion

Binge alcohol exposure, resulting in BALs of ~250mg/dL, produced reversible changes in indices of white matter microstructural integrity of selective fiber tracts: the genu and fimbria-fornix of the hippocampus were affected (FA was substantially lower in genu and fimbria-fornix systems, and MD was higher in fimbria-fornix), while the splenium was spared. The directionality of FA and MD changes comports with findings in chronically drinking alcoholic humans showing a focal vulnerability of anterior white matter fiber systems, quantified as lower FA and higher MD in cross-sectional study [7,8,11,23–27] and longitudinal follow-up [12,56]. We also report changes in λ_L_ and λ_T_: in the genu, λ_L_ was lower following binge alcohol treatment and returned to baseline levels with recovery; in the fimbria-fornix, λ_L_, and λ_T_ were both higher with binge treatment and returned to baseline with recovery. Trends suggested that the lower FA was related to the cumulative effects of the binge treatments, i.e., total average BALs achieved during the 4 days of binge alcohol exposure. To our knowledge, this is the first demonstration of a relationship between BALs and brain DTI metrics.

A potential limitation of the current study is that the DTI changes observed as a consequence of binge alcohol treatment are the result of partial volume effects of quantification of fiber pathways near the ventricles, which our previous study noted were enlarged [35]. Two results mitigate against that conclusion. Firstly, if partial volume effects were responsible for the quantified DTI changes in response to binge alcohol, both the genu and the splenium, structures similarly adjacent to the ventricles, should have shown altered DTI metrics. Secondly, the fiber tracking results were corroborated by TBSS, which is less prone to partial voluming effects [57] and showed that the genu, but not the splenium responded to binge alcohol exposure. Another potential limitation is that forced alcohol exposure is a simple method to evaluate questions regarding the effects of high alcohol levels on neuropathology but does not model free-choice drinking. High alcohol drinking in AUD is mediated by complex neurobiological, environmental, and behavioral interactions, and animal models of free-choice drinking have greater validity than forced exposure [58]. However, an alcohol deprivation effect model used in alcohol preferring (P rat) [59,60] and in high alcohol drinking (HAD rat) lines [61,62] demonstrates free-choice drinking to BALs of only 80mg% and a drinking in the dark, multiple scheduled access procedure also using selectively bred rats (P and HAD rats) similarly achieves BALs approaching only 80mg% [63,64]; thus, free choice drinking in rodent models fails to approach levels frequently achieved by heavy drinkers (>200mg%) [65–70]. Finally, ideally, isotropic acquisition is the best approach. However, signal-to-noise constraints make this impractical so the standard compromise is to have higher resolution in-plane than through- plane and to recognize that the 3D reformatting has this limitation

Recent evidence from both human and animal studies indicates the potential for rapid changes in DTI-derived measures [71–73]. Here, the genu in response to binge alcohol exposure showed a pattern of significantly reduced FA and λ_L_ that are similar to results in a mouse brain trauma model where FA and λ_L_ were both significantly reduced following trauma induction, the extent of which correlated with *in vitro* indices of axonal injury [74,75]. Although the results herein do not specifically demonstrate axonal disruption, alcohol is known to disrupt microtubules of cerebellar granule cell axons and Purkinje cell dendrites (3 months alcohol, sole source of fluid)[76] and axons in the extrapyramidal system, mesolimbic system, and several hypothalamic nuclei (16 months free-choice alcohol, alcohol-preferring rats)[77]. Thus, the effects of alcohol on DTI metrics in the genu could be interpreted as representing axonal injury.

The fimbria-fornix demonstrated reduced FA and elevated MD, λ_L_, and λ_T_ during binge alcohol treatment. This pattern of changes is consistent with those observed in a transgenic rodent model: myelin-deficient jimpy mice have profound astrocytic hypertrophy and abnormally low FA and high MD, λ_L_, and λ_T_ [78]. High alcohol exposure results in hypertrophied astrocytes in the hippocampus in mice (5 days alcohol, sole source of fluid) [79], and hippocampal astrogliosis, as quantified by immunohistochemical staining for vimentin in rats (4 days alcohol, intragastric) [80]. Thus the effects of alcohol on DTI metrics in the fimbria-fornix could be interpreted as representing astrocytic hypertrophy.

In conclusion, acute, quickly achieved, high doses of alcohol result in DTI-detectable effects on white matter that, although transient, corroborate findings in human alcoholics, demonstrating an anterior distribution of disrupted white matter fiber tracts with change toward normality with sobriety [12].
